# Global biodiversity data suggest allopolyploid plants do not occupy larger ranges or harsher conditions compared with their progenitors

**DOI:** 10.1002/ece3.10231

**Published:** 2023-08-17

**Authors:** Julia K. Mata, Sara L. Martin, Tyler W. Smith

**Affiliations:** ^1^ Agriculture and Agri‐Food Canada Ottawa Ontario Canada

**Keywords:** adaptation, allo, climatic range, genome duplication, geographic range, polyploid

## Abstract

Understanding the factors determining species' geographical and environmental range is a central question in evolution and ecology, and key for developing conservation and management practices. Shortly after the discovery of polyploidy, just over 100 years ago, it was suggested that polyploids generally have greater range sizes and occur in more extreme conditions than their diploid congeners. This suggestion is now widely accepted in the literature and is attributed to polyploids having an increased capacity for genetic diversity that increases their potential for adaptation and invasiveness. However, the data supporting this idea are mixed. Here, we compare the niche of allopolyploid plants to their progenitor species to determine whether allopolyploidization is associated with increased geographic range or extreme environmental tolerance. Our analysis includes 123 allopolyploid species that exist as only one known ploidy level, with at least one known progenitor species, and at least 50 records in the Global Biodiversity Information Facility (GBIF) database. We used GBIF occurrence data and range modeling tools to quantify the geographic and environmental distribution of these allopolyploids relative to their progenitors. We find no indication that allopolyploid plants occupy more extreme conditions or larger geographic ranges than their progenitors. Data evaluated here generally indicate no significant difference in range between allopolyploids and progenitors, and where significant differences do occur, the progenitors are more likely to exist in extreme conditions. We concluded that the evidence from these data indicate allopolyploidization does not result in larger or more extreme ranges. Thus, allopolyploidization does not have a consistent effect on species distribution, and we conclude it is more likely the content of an allopolyploid's genome rather than polyploidy per se that determines the potential for invasiveness.

## Abstract 2

Understanding the factors shaping a species' geographical and environmental range is a central question in evolution and ecology, and polyploidization has been cited as a process that results in tolerance to a larger range of or more extreme conditions. Here, we compare the niche of 123 allopolyploid plants to their progenitor species to determine whether allopolyploidization is associated with this type of change using records in the Global Biodiversity Information Facility database. We find no indication that allopolyploid plants occupy more extreme conditions or larger geographic ranges than their progenitors.

## 1 INTRODUCTION

A key question in evolution and ecology is what factors shape the geographic range limits of species (Brown et al., 1996; Gaston, 1996; Sheth et al., 2020). Many studies have investigated the factors that influence a plant species' range and abundance, including anthropogenic effects, dispersal dynamics, and biotic interactions. Two major, potentially interacting factors are phylogeny and ploidy (e.g., Ficetola & Stöck, 2016; Gaynor et al., 2018; Martin & Husband, 2009; Pandit et al., 2011). This area of research has practical applications in conservation and management efforts as we attempt to understand which species are likely to remain rare or become highly invasive.

Species that can adapt to novel environments are more likely to become invasive and genome duplication may provide the raw material to enable this adaptation (Galbraith et al., 2011; Levin, 2000; Otto & Whitton, 2000; Stebbins, 1985; te Beest et al., 2012; Van de Peer et al., 2017). Genome duplication events create species with more than two complete genome copies (polyploids), and in the case of allopolyploids, combine the genomes from two or more differentiated lineages (Ramsey & Schemske, 1998). This can result in species with greater genetic diversity, shifts in genetic systems, altered phenotypes, and increased heterozygosity (Otto & Whitton, 2000; Soltis & Soltis, 2000; Stebbins, 1985). The amount of variation incorporated in a polyploid genome has the potential to be greater than that of its progenitor genomes, particularly when the new species has multiple origins (reviewed by Soltis & Soltis, 1999), or recurrent gene flow with progenitors (Cheng et al., 2019; Schmickl & Yant, 2021; Wang et al., 2020). Further, redundancy in the genome can provide additional flexibility allowing for the divergence of gene regulation, expression, and function (reviewed by Adams, 2007, Nieto Feliner et al., 2020). Together these changes may lead to a greater potential to adapt to different conditions, and allopolyploidization is associated with the occupation of more extreme conditions in some taxa (e.g., Ficetola & Stöck, 2016). It is often broadly asserted that polyploids occupy more extreme conditions than their progenitors, and consequently occur at higher elevations and in more poleward locations as a result (e.g., Levin, 2000; Rice et al., 2019).

Shifts in the genetic systems and phenotypes in polyploids have also been associated with a greater range size. Allopolyploidization has been found to lead to larger geographic ranges in the fish family Cyprinidae (Li & Guo, 2020), and in plants such as *Arabidopsis kamchatica* (Paape et al., 2020), *Trifolium repens* L. (Griffiths et al., 2019), and tetraploid derivatives of *Aegilops* (Huynh et al., 2020). Additionally, within different cytotypes of *Crataegus* subgenus *Sanguineae*, autopolyploid and diploid congeners had smaller range sizes when compared to allopolyploids (Coughlan et al., 2017). More broadly, a study assessing the influence of ploidy on invasiveness in plants found that vulnerability to endangerment is more common for diploid plants and invasiveness is more likely for polyploids (Gaynor et al., 2018).

However, while examples of allopolyploid species occupying harsher conditions and larger ranges than their progenitors have been found, it is not clear that this is a general consequence of allopolyploidy, and many counterexamples at different scales are also available. For example, a reciprocal transplant experiment on five cytotypes from the *Claytonia perfoliata* complex, where the diploid *C. rubra* is found at the highest elevation, concluded that polyploids did not have an overall advantage (McIntyre & Strauss, 2017). Less extreme climatic tolerances, narrower climatic tolerances or geographical range for allopolyploids compared with related diploids or “unexpected” occurrence of the polyploids at lower elevation has been demonstrated for European primroses (Theodoridis et al., 2013), *Mercurialis annua* L. (Buggs & Pannell, 2007), *Gymnosphaera metteniana* (Hance) Tagawa (Wang et al., 2020), *Festuca amethystina* L. (Kiedrzynski et al., 2021) and species within the genus *Isoetes* (Liu et al., 2004). Additionally, genera such as *Solanum*, or the previously mentioned *Claytonia*, which have been the subject of more thorough investigation at the genus level, reveal complexity in the characteristics of diploid and polyploid members (Hijmans et al., 2007).

At a macroecological scale, recent studies have also found no evidence that polyploids have larger geographic or climatic ranges than diploids (Kelly & Woodward, 1996; Martin & Husband, 2009; Sheth et al., 2020; Stebbins & Dawe, 1987). However, in these previous macroecological studies, the resolution of the data was limited, for example when described by occurrence within political jurisdictions, or restricted to a specific region or flora. This may overestimate the range of climatic conditions attributed to species while limiting the potential range size or climatic condition variation seen between the polyploids and their diploid congeners (Kirchheimer et al., 2016). These studies also did not attempt to separate autopolyploids and allopolyploids or use their progenitors for comparison. There are likely strong differences in the consequences of these two processes because of the role of characteristics such as tetrasomic inheritance patterns in shaping autopolyploid lineages (Bomblies, 2022; Otto & Whitton, 2000) and the role of hybridization in shaping allopolyploid lineages. More crucially for understanding the evolutionary consequences of these processes, direct comparisons to diploid progenitors are essential to asking whether allopolyploidization per se is contributing to the differences observed among species, or if the perceived impact of this process is due to limited data or confounding factors such as phylogenetic conservatism.

Our investigation asks whether allopolyploidy facilitates the colonization of larger or more climatically extreme ranges compared with related diploids, rather than whether allopolyploids occur more frequently in these environments. This is a critical distinction as the frequency of allopolyploids in a particular environment may reflect the prevalence of polyploids in the clades that occur there, rather than any inherent adaptive capacity conferred by allopolyploidization. On the contrary, if allopolyploids are shown to have consistently broader ranges than their diploid relatives, it will provide much stronger evidence for the general adaptive value of allopolyploidization. Here, we use herbarium records and climate data to contrast the geographic and environmental distribution of 123 allopolyploids to their diploid progenitors, as a direct test of the hypothesis that allopolyploids occupy larger and more environmentally extreme distributions from the diploids they are descended from.

## 2 MATERIALS AND METHODS

### 2.1 Species selection

We selected species by reviewing the scientific literature to determine a list of known allopolyploid plant species (Table S1). Plant species that had at least one known progenitor species were recorded; however, those with multiple levels of ploidy were not considered because it was not possible to determine the ploidy of the specimens in the Global Biodiversity Information Facility (GBIF) database. We determined whether a species had more than one cytotype by reviewing the available literature for that species. Some allopolyploid species had progenitor species that were also polyploids. We completed our analyses for the complete dataset, and also on the subset of species groups with diploid progenitors, so that we could compare diploids to their polyploid (usually tetraploid) derivatives and polyploids to higher‐ploidy derivatives (e.g., tetraploids to their octoploid derivatives).

There was one special case when selecting species. For *Capsella bursa‐pastoris*, we included *Capsella rubella* and *Capsella grandiflora* as parents because *C. bursa‐pastoris* shows signs of admixture with both species but did not include *C. orientalis* because of a lack of records (Douglas et al., 2015; Kryvokhyzha et al., 2019).

### 2.2 Data collection

After synthesizing a list of species, we downloaded the available occurrence data from GBIF for the species and their progenitors (GBIF.org, 2020). We collected occurrence data from one allopolyploid and its progenitor species for 123 species from 43 different genera. Only the preserved specimen data were used by selecting “Preserved specimen” under the “Basis of record” tab. We excluded species with fewer than 50 GBIF records, to avoid rarely collected and poorly documented taxa.

### 2.3 Quantifying distributions

We quantified the geographic and climatic distribution of each species, with variables selected to capture the extremity (i.e., maximum or minimum values) and breadth along each gradient.

Geographically, we quantified the position of each species using both the maximum latitude and the latitude of the range centroid (center of mass or mean latitude). In both cases, we used the absolute latitude value (i.e., ignoring the North or South direction), to reflect the relative poleward position of the species. We measured geographic range as the distance (in degrees) between the northern‐most and southern‐most records. We quantified the geographic area as “area of occupancy” (sensu IUCN 2012) by applying a 10 km buffer around each presence point and calculating the total area of the buffers that existed on land for each species. We rejected other area measures, such as minimum convex hulls, as it was evident for many species that such values would include large areas of oceans or other inappropriate habitats.

**FIGURE 1:**
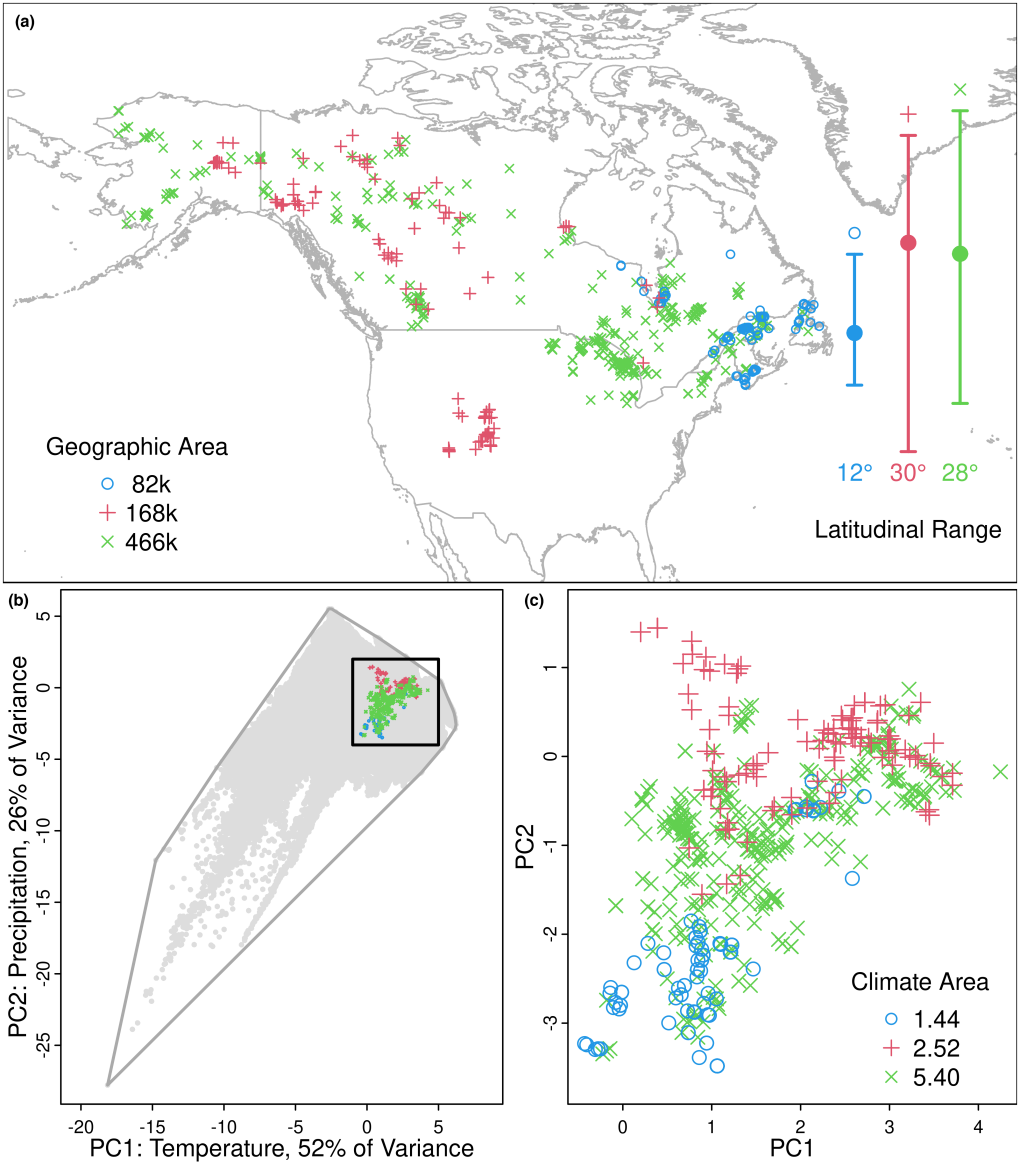
Geographic and climatic distribution of the allotetraploid *Primula laurentiana* (blue circles), and its putative progenitors *P. incana* (red +) and *P. mistassinica* (green x). (a) Geographic distribution of GBIF herbarium records. The latitudinal range is displayed on the right as the distribution minimum, maximum, and mean, with the absolute range listed below. The geographic area of occupancy for each species is listed in the lower left (calculation described in the text). (b) Principal Components Analysis ordination of the global climate data. The gray points indicate the position of each raster cell in the global Worldclim dataset. The colored symbols are the climate conditions for the records of each of the three taxa, using the same symbols as panel (a). (c) The same ordination enlarged to show the relative positions of the three taxa. The climate occupancy (calculation described in the text) is listed in the lower right.

This process is illustrated in Figure 1a, which displays the GBIF records for the octoploid *Primula laurentiana* Fernald and its putative progenitors, the hexaploid *P. incana* M. E. Jones and the diploid *P. mistassinica* Michaux (Guggisberg et al., 2006). The latitude maximum, mean and range are taken directly from the coordinate records, as shown. A 10 km buffer around each point is used to calculate the geographic area of occupancy; these squares are too small to visualize on a map of this scale, but the values match expectations based on a visual assessment of the map: *Primula laurentiana* has the smallest range, assessed as 28,000 km^2^, compared with *P. mistassinica*, which has the largest range (145,000 km^2^).

We used WorldClim data (Fick & Hijmans, 2017) at 10‐min resolution to assess temperature and moisture gradients. For temperature, we assessed the highest monthly maximum (BIO5), lowest monthly minimum (BIO6), and annual range (the difference between the two) for each species. Similarly, for moisture, we assessed the highest monthly maximum precipitation (BIO13), the lowest monthly minimum precipitation (BIO14), and precipitation seasonality (i.e., coefficient of variation for monthly precipitation, BIO15).

We also calculated two multivariate measures of the climatic breadth of each species, using all 19 WorldClim bioclimatic variables (Fick & Hijmans, 2017). For the first, we performed a principal component analysis (PCA) to reduce the 19 bioclimatic variables into two dimensions and divided this space into a grid with a resolution of 0.2 units (total extent 122 × 167 ≈ 20,000 cells). We then projected the occurrences of each species into this space using the function “suprow” from the package ade4 (Thioulouse et al., 2018) and calculated their environmental range area as the number of grid cells they occupied. Thus, the environmental area of all species was calculated on the same scale. We refer to this measure as climate occupancy.

This process is illustrated in Figure 1b,c. Climate space is defined by the first two axes of a PCA calculated from the 19 variables for each raster cell in the global WorldClim data, a total of 584,521 observations. The first two axes captured 56% and 26% of the total variation, respectively (Figure 1b). The variables with the highest loadings on PC1 were temperature measures, while the variables correlated with PC2 were mostly precipitation measures (Table 1).

**TABLE 1:** Global climate PCA variable loadings.

Axis 1		Axis 2	
Variable	Loading	Variable	Loading
Bio6: Minimum temperature of coldest month	−0.96	Bio17: Precipitation of driest quarter	−0.80
Bio11: Mean temperature of coldest quarter	−0.96	Bio14: Precipitation of driest month	−0.80
Bio1: Annual mean temperature	−0.93	Bio2: Mean diurnal temperature range	0.73
Bio3: Isothermality (daily temp range/annual temp range)	−0.91	Bio12: Annual precipitation	−0.68
Bio4: Temperature seasonality	0.90	Bio19: Precipitation of coldest quarter	−0.62

*Note*: The top five variables for PC axis 1 and 2 ordered by their absolute loading on each axis.

The same global climate space was used for all species in our analyses. The 19 variables are extracted for each record in the GBIF data, which we then projected onto the global climate PCA. In the case of *Primula laurentiana* et al., the three species plotted together in the upper right quadrant of the global PCA (Figure 1b). Enlarging that region (Figure 1c) reveals that *P. laurentiana* occupies a visually smaller range of environments than either of its putative progenitors, an observation, which is corroborated by the corresponding environmental areas of 1.44 for *P. laurentiana*, compared with 2.52 and 5.40 for the two progenitors.

**FIGURE 2:**
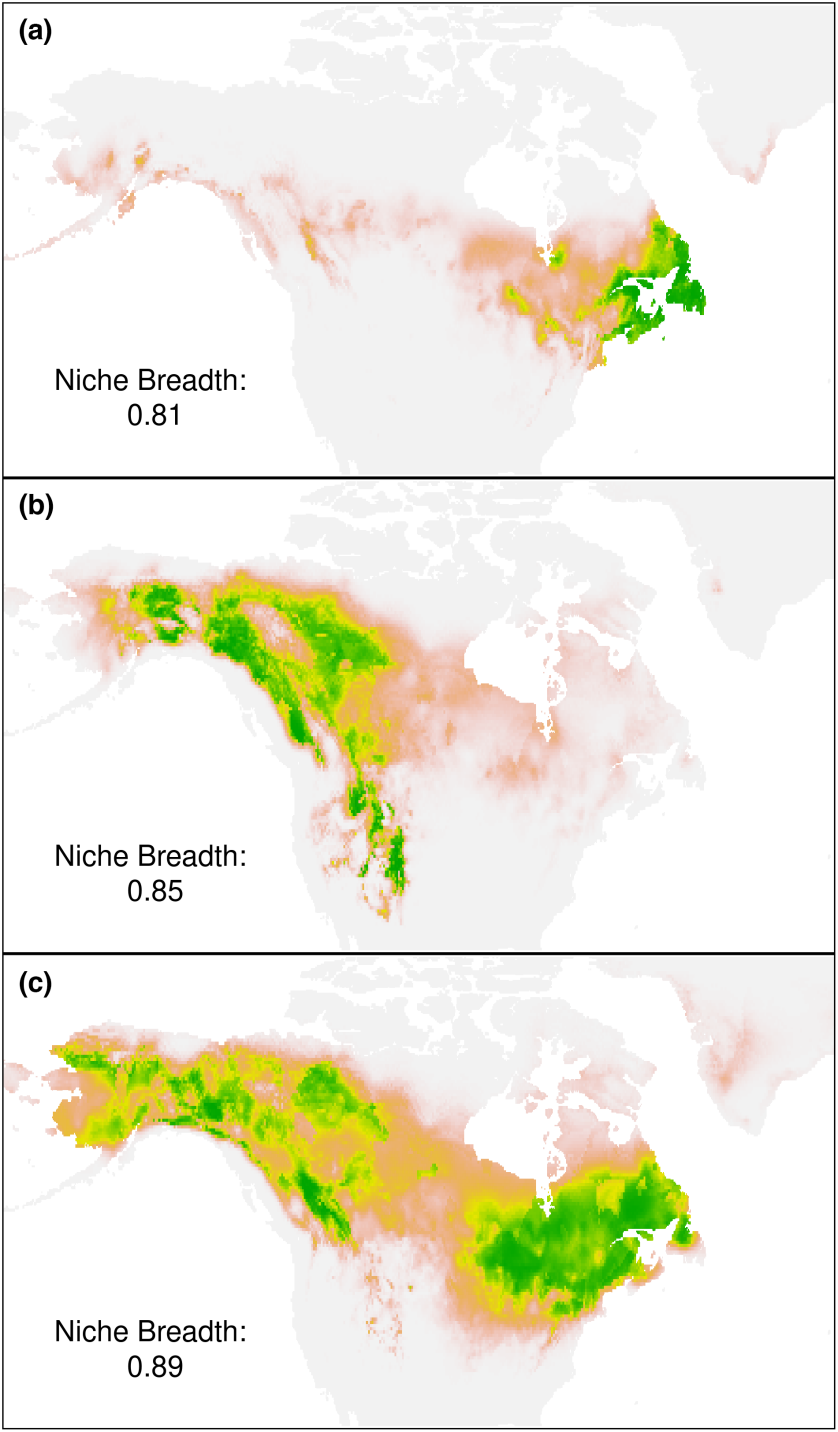
Maxent distribution models for the allotetraploid *Primula laurentiana* (a), and its putative progenitors *P. incana* (b) and *P. mistassinica* (c). The colors on each map indicate projected habitat suitability, with highest suitability in green, declining through yellow and orange. The niche breadth calculated from each distribution model is indicated in the lower left.

For the second multivariate climate index, we used Maxent distribution models (Phillips et al., 2017). Our geographic and environmental analyses capture the spatial and climatological distribution of the species. Maxent combines both aspects in a single model, quantifying the environmental suitability of geographic locations for a species. As Maxent accounts for collinearity among variables (Feng et al., 2019), we used all 19 WorldClim bioclim variables (Fick & Hijmans, 2017). Unlike the previously described analyses, Maxent is sensitive to the density of observations (Radosavljevic et al., 2014). To account for this potential bias, we thinned the records using the grid sampling method of Hijmans et al. (2017). We set the grid resolution to 1 min and retained only one record per cell for inclusion in our analysis. To ensure all models could be compared on the same scale, we set the background study extent to the full WorldClim raster layers for all species. We quantified the breadth of the niche models using Levins (1968) niche breadth metric (B1), calculated with the “raster.breadth” function of the ENMTools package. As an example, niche models and niche breadth values for *P. laurentiana* et al. are presented in Figure 2.

In addition to the distribution measures, we also calculated the geographic and climate overlap between each allopolyploid and its progenitors, and between the two progenitors. For geographic overlap, we compared the area occupied by each species using the function “geog.range.overlap” from the “ENMTools” package (Warren & Dinnage, 2021). This measure ranges from 0 (no overlap between species) to 100 (the smaller range falls completely within the larger range).

We used niche stability, calculated with the function “niche.clim.dyn.index” in the ecospat R package, to quantify the environmental overlap between species. The environment is quantified using the same PCA axes as for the environmental area. Niche stability is the proportion of the environmental space occupied by the allopolyploid that is also occupied by its diploid parent.

### 2.4 Statistical analysis

To compare the geographic and environmental distribution of diploid and polyploid species, we scored the relative value of each assessed variable for each polyploid as the most extreme, least extreme, or intermediate between the values of its diploid congeners. We tallied the total number of polyploid species in each category and compared these counts to the null expectation of an equal number appearing in each category with a Chi‐squared test (contingency analysis). If ploidy has no influence on distribution, we expect equal numbers of polyploids in each category. On the contrary, if polyploidy leads to larger or more extreme ranges, they should be over‐represented in the “most extreme” category when compared to their progenitors.

We completed this test for all allopolyploids in our dataset with two known parents (*n* = 71). We also tested subsets of this data limited to only seed plants (*n* = 55), and only ferns and lycophytes (*n* = 16), to see whether there were different patterns in these groups. In all cases, we completed separate analyses for all allopolyploids, and for only those polyploids with diploid progenitors (*n* = 66, i.e., excluding five allopolyploids derived from polyploid progenitors). We considered further sub‐categories (i.e., Asteraceae, Poaceae, Solanaceae), but there were too few species in these groups to support the analysis.

Finally, we completed a separate set of analyses for allopolyploids for which only one parent was known (*n* = 52). In this case, we scored the allopolyploid as being either more or less extreme than its known parent for each variable and modified the Chi‐square test accordingly. To account for multiple testing, we set the statistical significance value for each set of tests (with 12 assessed variables) at the Bonferroni‐adjusted value of 0.05/12 = 0.0042.

We tested the significance of the geographic and environmental range overlap between the allopolyploids and their progenitors, compared with the overlaps between the two progenitors, using a pairwise Welch's *t*‐test, using the “t.test” function in R.

## 3 RESULTS

We found a total of 71 allopolyploids with two known parents and 52 with one known parent (Table S1). Of the 71 allopolyploid species with two known parents, only five had progenitors that were not diploid. We retrieved 322,402 records from GBIF, from 839 museums and herbaria (GBIF.org 2020).

Based on the Chi‐square tests for species with two known parents, only minimum precipitation is statistically different (*p* < .0001) from the results expected by chance. More specifically, allopolyploids are over‐represented in the intermediate category (Table 2). For the other eleven variables, there is no relationship between ploidy and distribution. The same is true when we remove the five allopolyploid species with polyploid progenitors (Table S2), and when we remove ferns and lycophytes and consider only seed plants (*n* = 55, Table S3). Restricting the analysis to ferns and lycophytes (*n* = 16), maximum precipitation (*p* < .001) was significantly different from the null expectation, with allopolyploids over‐represented in the least extreme category (Table S4).

**TABLE 2:** Ranking of 71 allopolyploids for 12 geographic and environmental range measures, as compared to their progenitors.

	Allopolyploid rank			*p*
	Least extreme	Intermediate	Most extreme	
Geography				
Maximum latitude	23	24	24	.986
Mean latitude	25	28	18	.329
Latitude range	27	18	26	.358
Area of occupancy	27	21	23	.674
Temperature				
Maximum	21	21	29	.406
Minimum	23	27	21	.674
Annual range	27	24	20	.594
Precipitation				
Maximum	28	16	27	.154
Minimum	19	**39**	13	**<.001**
Seasonality	30	20	21	.278
Climate				
Climate occupancy	27	18	26	.358
Maxent niche breadth	24	23	24	.986

*Note*: Values indicate the number of comparisons in which the allopolyploid was the least extreme, most extreme, or intermediate in value when compared to its two progenitors. *p* reports the *p*‐value from a Chi‐Square test for each variable. Bold numbers indicate statistically significant differences from the null expectation of equal distribution, assessed at the Bonferroni‐adjusted *p*‐value of .0042.

For species with only one known parent (*n* = 52), mean latitude, latitude range, minimum temperature, annual temperature range, precipitation seasonality, and Maxent niche breadth were all significantly different from null expectation (Table S5). For each variable, the allopolyploid was over‐represented in the least extreme category. The same pattern held when restricting the analysis to allopolyploids with diploid progenitors (*n* = 48, Table S6).

To protect against multiple comparisons generating false positives, we applied the Bonferroni correction to our statistical tests, considering each set of species as a separate family of 12 tests. This lowered the effective significance threshold for our tests to 0.05/12 = 0.0042. Consequently, our analysis may have generated false negatives. However, there are only 17 tests out of 120 that were not significant at an adjusted threshold of 0.0042 but that would have been significant at a 0.05 threshold (Table 2; Tables S2–S10). None of these comparisons show allopolyploids as more extreme than their progenitors. On the contrary, if we had considered all 120 tests as part of a single family of comparisons, our adjusted significance threshold would have been lowered to 0.0004, meaning even fewer significant differences, and still no support for allopolyploids having more extreme distribution than their progenitors. While there may be some questions as to the appropriate approach for dealing with multiple comparisons, our conclusions remain the same whether we ignore them, and use *p* = .05 (accepting the risk of inflated false positives), or, take the other extreme with *p* = .0004 (with an inflated risk of false negatives).

In summary, there was no variable for which allopolyploids were more extreme or had wider ranges than their progenitors, no matter which subset of the data we examined. Indeed, the statistically significant differences we did find all indicated that allopolyploids have more restricted distributions than their progenitors.

On average, there was a 23.9% (range 0–70.4%; SD 16.9) overlap in the geographic ranges of allopolyploids and their progenitors, compared with 16.0% (range 0–51.5%; SD 14.3) overlap between the two progenitors. This was a statistically significant difference (Welch's *t*‐test, *p* < .001). The environmental ranges of allopolyploids and progenitors overlapped on average 86.7% (range 10.2%–100%; SD 18.4), compared with 80.0% (range 14.8%–100%; SD 22.3) overlap between the progenitors. This was also statistically significant (Welch's *t*‐test, *p* = .005). The numerical results are nearly identical, and also statistically significant, when only diploid progenitors are considered.

## 4 DISCUSSION

Less than 30 years after the recognition that polyploid plant taxa existed, the idea that species with high chromosome numbers are often distributed in more extreme environments compared with those with a lower number had become established in the literature. This conclusion was based primarily on work by Hagerup and Tischler (Baquar, 1976; Müntzing, 1936) on studies of small regional floras: Timbuktu and Schleswig‐Holstein, a province in northern Germany (Tischler, 1935 cited by Baquar, 1976); and case studies with limited taxonomic scope (i.e., the genus *Danthonia*, Hagerup, 1939). This conclusion was challenged by researchers performing common garden experiments comparing the performance of different cytotypes (Bowden, 1940; Clausen et al., 1940, 1945). Further, other flora‐based studies such as Baquar's (1976) study of the Flora of Pakistan did not find similar patterns nor have more recent floristic studies (Kelly & Woodward, 1996; Martin & Husband, 2009; Petit & Thompson, 1999; Stebbins & Dawe, 1987). However, the idea that polyploidy per se is responsible for invasive, weedy, large ranges or adaptation to particular environmental conditions has remained an appealing explanation frequently invoked as a general explanation for the geographic and environmental distributions.

Here, we used worldwide occurrence data and range modeling to test whether 123 allopolyploids have a greater or more extreme environmental and geographical range than their progenitor species. Using museum specimen records, we were able to take a global perspective, with finer resolution and broader taxonomic coverage than previous studies, and explicitly contrast the distribution of allopolyploids with their diploid progenitors. We find that measured across a wide variety of geographic and environmental axes, allopolyploids show no consistent differences in distribution relative to their progenitors. Indeed, allopolyploids often had less extreme climatic variables than their progenitors and greater overlap with these progenitors than the progenitors had with each other. We compared allopolyploids with their progenitors for 12 environmental and geographic variables for species with two known parents, one known parent and then repeated the analysis excluding polyploid parents, and analyzed ferns and lycophytes separately from seed plants, for a total of 120 comparisons. In no case did we find allopolyploids were more likely to have a more extreme or broader range than their progenitors. In four comparisons, the allopolyploid was more likely to be intermediate to their two progenitors, and in 25 comparisons they were actually more likely to be the least extreme when compared to their progenitors. While these are not 120 independent tests, due to the nesting of the various subsets of the data, it is clear there is no support for the hypothesis that allopolyploids have a broader range than their progenitors. Similarly, in cases with only one known parent where we are limited to examining half of the progenitor‐descendant relationship, in every comparison, the allopolyploids were less extreme in their distributions than their single known progenitor (Tables S7–S10). While we cannot know the proportion of these comparisons in which the allopolyploids were over‐represented in the least extreme category, rather than the intermediate category, we can be certain that they were never over‐represented in the most extreme category leading to the same conclusion: There is no support for the hypothesis that allopolyploids have a broader range than their progenitors.

Given the broad scope of our study, it was not possible to personally collect the data necessary for our analysis. We were dependent on publicly available data in the GBIF repository. This allowed us to include more than 100 allopolyploids, and nearly twice as many progenitor species, and quantify and compare their distributions using hundreds of thousands of observations from hundreds of institutions. However, this also means our analysis was premised on the assumptions that the coordinates for these records are accurately recorded, and the specimens are correctly and consistently identified. Clearly, this is not true; nor, given the quantity of specimens involved, were we able to directly assess the extent to which these data included inaccuracies.

While this problem is a consequence of the volume of data we are working with, that volume also provides a partial resolution. Another, often unwritten, assumption of such large‐scale macroecological analyses is that errors in the data are few relative to the total quantity of records employed (Atwater, 2021; Häkkinen et al., 2021; Rice et al., 2019). While some of the records are erroneous, and perhaps some of the species comparisons in our study have yielded incorrect results as a consequence, we assume the influence of such errors are small relative to the total analysis. While this may not allay all concerns regarding our source data, we think our approach, combining the direct comparison of polyploids to their progenitors, on a global scale, and with extensive taxonomic coverage, is an important perspective on an issue that is frequently addressed in a much more granular way and often with this same data.

While our study does not directly answer whether polyploids increase in frequency with latitude, we do not see a consistent shift to higher latitudes, measured as either the range centroid or the maximum absolute latitude, in allopolyploids compared with their diploid progenitors. Again, the relationships that are significant suggest the opposite, with progenitors in more extreme positions. This suggests that allopolyploids are not more likely to occupy higher latitudes compared with their diploid progenitors and a poleward shift is not a general result of allopolyploidization. This contradicts the conclusions of Rice et al. (2019), who also used GBIF occurrence data but more broadly looked at the global distribution of polyploids compared with all diploids. Evidence suggesting polyploids increase away from the equator, as in Rice et al.'s (2019) study, may be biased by the frequency of polyploid‐rich taxonomic groups and lifeforms in these areas and, perhaps more significantly, by the lack of cytological and taxonomic research completed in the tropics. Genome sizes are available for only about 1%–2% of angiosperms; with woody species and tropical species poorly represented (Galbraith et al., 2011). This poor representation of woody plants in polyploidy research may also contribute to Rice et al. (2019) results showing a high frequency of polyploids with perennial herbs and a low frequency for woody species. Alternatively, polyploid range may not be directly related to increasing latitude but instead to related available opportunities for colonization (Brochmann et al., 2004).

These results provide further evidence that, despite frequent claims, polyploidization does not influence a species range in a consistent way. There is evidence supporting that polyploidization contributes to speciation as is indicated by the common pattern that large‐scale species radiations occur after polyploidization events (Schranz et al., 2012; Soltis et al., 2009) and the ubiquity of an evolutionary history including polyploidization in plants (Blanc & Wolfe, 2004; Murat et al., 2017). Thus, polyploidization can contribute to divergence and result in the establishment of a new species with a distinct range size, ecological tolerance, dispersal mechanism, or mating system. Indeed, the niches of allopolyploids may diverge more quickly than diploids (Baniaga et al., 2020). However, due to shared evolutionary history and similar genetics, polyploids and their diploid progenitor species are expected to have similarities in their geographical and climatic range (Pyron et al., 2015). These similarities likely account for the high climatic overlap between allopolyploids and their progenitors, 87%, which was slightly, but significantly, greater than the overlap between progenitors (80%). While the geographic overlap between allopolyploids and their progenitors was lower (24%), it was also significantly greater than the overlap between progenitors (14%). A similar finding following a detailed examination of the niches of 14 allopolyploids in comparison with their progenitors was reported by Marchant et al. (2016). These allopolyploids showed strong overlap with at least one progenitor and most frequently showed niche intermediacy—that is—niche characteristics between the two progenitors. This suggests that allopolyploids' range size is strongly influenced by the climatic and geographical range characteristics of the progenitor species as has been found in detailed studies of allotetraploid *Aegilops* species by Huynh et al. (2020). The degree of overlap may also be dependent on the time since speciation as older genome duplication events will have had more time to diverge from their progenitors, but it is clear that in some cases, the niche divergence may be more subtle than in others (Marchant et al., 2016; Ramsey & Schemske, 1998; Weiss‐Schneeweiss et al., 2013).

Certainly, some allopolyploid species have greater or more extreme climatic and larger geographical ranges than their progenitors (e.g., *Aegilops cylindrica*) and several factors likely contribute to the success of these species, such as derivation from repeated or multiple origins (Meimberg et al., 2009) or strong dispersal abilities (Glennon et al., 2014). However, the conclusion of this and similar studies is that it is not a consistent pattern attributable to allopolyploidization nor is there a consistent direction of these changes, for example to greater drought or cold tolerance. McIntyre and Strauss (2017) suggested that because range size varies for polyploids within genera, the polyploids with large ranges have been more likely to be sampled than those with smaller, more restricted ranges. This may have contributed to the conclusions reached in the very earliest days of studying polyploidy (Müntzing, 1936).

Our knowledge of polyploidy has grown substantially over the last 100 years, but we still have only examined a small portion of the world's flora and only a small fraction of polyploid species have known mode of origin or progenitors. Future work needs to address the biases in our knowledge of polyploids, which lacks information on tropical and woody species as highlighted by Galbraith et al. (2011), to determine how our sampling of taxa has influenced our conclusions. Further, to isolate the effects of polyploidy and hybridization per se, researchers would ideally compare the geographic and climatic ranges of three types of species and their progenitors: autopolyploid, allopolyploidy, and homoploids (Ramsey & Schemske, 1998; Rieseberg, 1997). However, more examples of autopolyploidy or homoploids and their progenitors with information on their geographic ranges need to be documented for this type of analysis to be possible (Abbott et al., 2010; Rieseberg, 1997; Soltis et al., 2007; Spoelhof et al., 2017). At this point, however, the balance of the evidence suggests that the genes and the combinations of genes captured within the allopolyploid matter more for determining the species' climatic tolerance, geographic range, and ultimately their probability of becoming weedy or invasive than allopolyploidization itself.

## AUTHOR CONTRIBUTIONS

**Julia K. Mata:** Conceptualization (supporting); data curation (equal); formal analysis (equal); investigation (equal); methodology (supporting); writing – original draft (equal); writing – review and editing (equal). **Sara L. Martin:** Conceptualization (equal); formal analysis (supporting); funding acquisition (equal); investigation (equal); methodology (equal); resources (equal); supervision (equal); writing – original draft (equal); writing – review and editing (equal). **Tyler W. Smith:** Conceptualization (equal); formal analysis (equal); funding acquisition (equal); investigation (equal); methodology (equal); resources (equal); supervision (equal); writing – original draft (equal); writing – review and editing (equal).

## FUNDING INFORMATION

This project was funded by Agriculture and Agri‐Food Canada (project J‐002275).

## Supporting information


Table S1

Tables S2–S10

## Data Availability

The raw data used in this study are available from GBIF through the following links: doi.org/10.15468/dl.gtmpgj, doi.org/10.15468/dl.8b5tde doi.org/10.15468/dl.mv6m97, doi.org/10.15468/dl.pex9un, doi.org/10.15468/dl.8eb9af, doi.org/10.15468/dl.3c4a6v, doi.org/10.15468/dl.5kmean, doi.org/10.15468/dl.p42dg4, doi.org/10.15468/dl.drnj7s. If the manuscript is accepted, then the code for the analysis will be available at datadryad.org.
