# Supplementary material for: Global biodiversity data suggest allopolyploid plants do not occupy larger ranges or harsher conditions compared with their progenitors
Source: Ecol Evol. 2023 Aug 17;13(8):e10231. doi: 10.1002/ece3.10231 (PMC10433117; doi:10.1002/ece3.10231)
Supplement: Supplementary file 2 — Tables S2–S10 [file ECE3-13-e10231-s002.docx]

Table 1

|  | Allopolyploid Rank | | |  |
| --- | --- | --- | --- | --- |
|  | Least Extreme | Intermediate | Most Extreme | P |
| Geography | | | | |
| Maximum latitude | 23 | 24 | 24 | 0.986 |
| Mean latitude | 25 | 28 | 18 | 0.329 |
| Latitude range | 27 | 18 | 26 | 0.358 |
| Area of Occupancy | 27 | 21 | 23 | 0.674 |
| Temperature | | | | |
| Maximum | 21 | 21 | 29 | 0.406 |
| Minimum | 23 | 27 | 21 | 0.674 |
| Annual Range | 27 | 24 | 20 | 0.594 |
| Precipitation | | | | |
| Maximum | 28 | 16 | 27 | 0.154 |
| Minimum | 19 | **39** | 13 | **< 0.001** |
| Seasonality | 30 | 20 | 21 | 0.278 |
| Climate | | | | |
| Climate Occupancy | 27 | 18 | 26 | 0.358 |
| Maxent Niche Breadth | 24 | 23 | 24 | 0.986 |

Table 1. Ranking of 71 Allopolyploids for 12 geographic and environmental range measures, as compared to their progenitors. Values indicate the number of comparisons in which the allopolyploid was the least extreme, most extreme, or intermediate in value, when compared to its two progenitors. P reports the P-value from a Chi-Square test for each variable. Bold numbers indicate statistically significant differences from the null expectation of equal distribution, assessed at the Bonferroni adjusted P-value of 0.0042.

Table S2 (Table S1 is in a separate file)

|  | Allopolyploid Rank | | |  |
| --- | --- | --- | --- | --- |
|  | Least Extreme | Intermediate | Most Extreme | P |
| Geography | | | | |
| Maximum latitude | 21 | 24 | 21 | 0.873 |
| Mean latitude | 23 | 26 | 17 | 0.385 |
| Latitude range | 25 | 18 | 23 | 0.554 |
| Area of Occupancy | 25 | 20 | 21 | 0.727 |
| Temperature | | | | |
| Maximum | 18 | 21 | 27 | 0.385 |
| Minimum | 20 | 27 | 19 | 0.422 |
| Annual Range | 24 | 24 | 18 | 0.594 |
| Precipitation | | | | |
| Maximum | 26 | 14 | 26 | 0.113 |
| Minimum | 17 | **38** | 11 | **< 0.001** |
| Seasonality | 28 | 19 | 19 | 0.293 |
| Climate | | | | |
| Climate Occupancy | 25 | 17 | 24 | 0.422 |
| Maxent Niche Breadth | 21 | 23 | 22 | 0.956 |

Table S2. Ranking of 66 Allopolyploids for 12 geographic and environmental range measures, as compared to their diploid progenitors (polyploid species with polyploid progenitors excluded). Values indicate the number of comparisons in which the allopolyploid was the least extreme, most extreme, or intermediate in value, when compared to its two progenitors. P reports the P-value from a Chi-Square test for each variable. Bold numbers indicate statistically significant differences from the null expectation of equal distribution, assessed at the Bonferroni adjusted P-value of 0.0042.

Table S3

|  | Allopolyploid Rank | | |  |
| --- | --- | --- | --- | --- |
|  | Least Extreme | Intermediate | Most Extreme | P |
| Geography | | | | |
| Maximum latitude | 17 | 15 | 23 | 0.389 |
| Mean latitude | 20 | 22 | 13 | 0.296 |
| Latitude range | 18 | 12 | 25 | 0.099 |
| Area of Occupancy | 16 | 17 | 22 | 0.569 |
| Temperature | | | | |
| Maximum | 17 | 15 | 23 | 0.389 |
| Minimum | 16 | 20 | 19 | 0.789 |
| Annual Range | 20 | 16 | 19 | 0.789 |
| Precipitation | | | | |
| Maximum | 14 | 15 | 26 | 0.089 |
| Minimum | 11 | **33** | 11 | **< 0.001** |
| Seasonality | 20 | 15 | 20 | 0.635 |
| Climate | | | | |
| Climate Occupancy | 16 | 14 | 25 | 0.154 |
| Maxent Niche Breadth | 14 | 20 | 21 | 0.458 |

Table S3. Ranking of 55 Allopolyploid seed plants (i.e., excluding ferns and lycophytes) for 12 geographic and environmental range measures, as compared to their diploid progenitors. Values indicate the number of comparisons in which the allopolyploid was the least extreme, most extreme, or intermediate in value, when compared to its two progenitors. P reports the P-value from a Chi-Square test for each variable. Bold numbers indicate statistically significant differences from the null expectation of equal distribution, assessed at the Bonferroni adjusted P-value of 0.0042.

Table S4

|  | Allopolyploid Rank | | |  |
| --- | --- | --- | --- | --- |
|  | Least Extreme | Intermediate | Most Extreme | P |
| Geography | | | | |
| Maximum latitude | 6 | 9 | 1 | 0.047 |
| Mean latitude | 5 | 6 | 5 | 0.939 |
| Latitude range | 9 | 6 | 1 | 0.047 |
| Area of Occupancy | 11 | 4 | 1 | 0.007 |
| Temperature | | | | |
| Maximum | 4 | 6 | 6 | 0.779 |
| Minimum | 2 | 7 | 7 | 0.210 |
| Annual Range | 7 | 8 | 1 | 0.068 |
| Precipitation | | | | |
| Maximum | **14** | 1 | 1 | **< 0.001** |
| Minimum | 2 | 6 | 8 | 0.174 |
| Seasonality | 10 | 5 | 1 | 0.022 |
| Climate | | | | |
| Climate Occupancy | 11 | 4 | 1 | 0.007 |
| Maxent Niche Breadth | 10 | 3 | 3 | 0.047 |

Table S4. Ranking of 16 Allopolyploid ferns and lycophytes for 12 geographic and environmental range measures, as compared to their diploid progenitors. Values indicate the number of comparisons in which the allopolyploid was the least extreme, most extreme, or intermediate in value, when compared to its two progenitors. P reports the P-value from a Chi-Square test for each variable. Bold numbers indicate statistically significant differences from the null expectation of equal distribution, assessed at the Bonferroni adjusted P-value of 0.0042.

Table S5

|  | Allopolyploid Rank | | |  |
| --- | --- | --- | --- | --- |
|  | Least Extreme | Intermediate | Most Extreme | P |
| Geography | | | | |
| Maximum latitude | 17 | 15 | 20 | 0.694 |
| Mean latitude | 18 | 21 | 13 | 0.390 |
| Latitude range | 18 | 12 | 22 | 0.232 |
| Area of Occupancy | 16 | 16 | 20 | 0.735 |
| Temperature | | | | |
| Maximum | 16 | 15 | 21 | 0.551 |
| Minimum | 15 | 20 | 17 | 0.694 |
| Annual Range | 19 | 16 | 17 | 0.874 |
| Precipitation | | | | |
| Maximum | 14 | 13 | 25 | 0.077 |
| Minimum | 10 | **32** | 10 | **< 0.001** |
| Seasonality | 20 | 14 | 18 | 0.584 |
| Climate | | | | |
| Climate Occupancy | 16 | 13 | 23 | 0.219 |
| Maxent Niche Breadth | 13 | 20 | 19 | 0.437 |

Table S5. Ranking of 52 Allopolyploid seed plants with diploid parents for 12 geographic and environmental range measures, as compared to their diploid progenitors. Values indicate the number of comparisons in which the allopolyploid was the least extreme, most extreme, or intermediate in value, when compared to its two progenitors. P reports the P-value from a Chi-Square test for each variable. Bold numbers indicate statistically significant differences from the null expectation of equal distribution, assessed at the Bonferroni adjusted P-value of 0.0042.

Table S6

|  | Allopolyploid Rank | | |  |
| --- | --- | --- | --- | --- |
|  | Least Extreme | Intermediate | Most Extreme | P |
| Geography | | | | |
| Maximum latitude | 4 | 9 | 1 | 0.030 |
| Mean latitude | 5 | 5 | 4 | 0.931 |
| Latitude range | 7 | 6 | 1 | 0.109 |
| Area of Occupancy | 9 | 4 | 1 | 0.030 |
| Temperature | | | | |
| Maximum | 2 | 6 | 6 | 0.319 |
| Minimum | 5 | 7 | 2 | 0.257 |
| Annual Range | 5 | 8 | 1 | 0.071 |
| Precipitation | | | | |
| Maximum | **12** | 1 | 1 | **< 0.001** |
| Minimum | 7 | 6 | 1 | 0.109 |
| Seasonality | 8 | 5 | 1 | 0.071 |
| Climate | | | | |
| Climate Occupancy | 9 | 4 | 1 | 0.030 |
| Maxent Niche Breadth | 8 | 3 | 3 | 0.168 |

Table S6. Ranking of 14 Allopolyploid ferns and lycophytes with diploid progenitors for 12 geographic and environmental range measures, as compared to their diploid progenitors. Values indicate the number of comparisons in which the allopolyploid was the least extreme, most extreme, or intermediate in value, when compared to its two progenitors. P reports the P-value from a Chi-Square test for each variable. Bold numbers indicate statistically significant differences from the null expectation of equal distribution, assessed at the Bonferroni adjusted P-value of 0.0042.

Table S7

|  | Allopolyploid Rank | |  |
| --- | --- | --- | --- |
|  | Least Extreme | Most Extreme | P |
| Geography | | | |
| Maximum latitude | 35 | 17 | 0.013 |
| Mean latitude | 37 | 15 | 0.002 |
| Latitude range | **39** | 13 | **< 0.001** |
| Area of Occupancy | 29 | 23 | 0.405 |
| Temperature | | | |
| Maximum | 25 | 27 | 0.782 |
| Minimum | **41** | 11 | **< 0.001** |
| Annual Range | **39** | 13 | **< 0.001** |
| Precipitation | | | |
| Maximum | 35 | 17 | 0.013 |
| Minimum | 27 | 25 | 0.782 |
| Seasonality | **37** | 15 | **0.002** |
| Climate | | | |
| Climate Occupancy | 33 | 19 | 0.052 |
| Maxent Niche Breadth | **40** | 12 | **< 0.001** |

Table S7. Ranking of 52 Allopolyploid plants with a single known progenitor for 12 geographic and environmental range measures, as compared to their progenitor. Values indicate the number of comparisons in which the allopolyploid was the least extreme or most extreme in value, when compared to its one known progenitor. P reports the P-value from a Chi-Square test for each variable. Bold numbers indicate statistically significant differences from the null expectation of equal distribution, assessed at the Bonferroni adjusted P-value of 0.0042.

Table S8

|  | Allopolyploid Rank | |  |
| --- | --- | --- | --- |
|  | Least Extreme | Most Extreme | P |
| Geography | | | |
| Maximum latitude | 33 | 15 | 0.009 |
| Mean latitude | 37 | 11 | < 0.001 |
| Latitude range | **35** | 13 | **0.001** |
| Area of Occupancy | 25 | 23 | 0.773 |
| Temperature | | | |
| Maximum | 22 | 26 | 0.564 |
| Minimum | **37** | 11 | **< 0.001** |
| Annual Range | **36** | 12 | **0.001** |
| Precipitation | | | |
| Maximum | 31 | 17 | 0.043 |
| Minimum | 26 | 22 | 0.564 |
| Seasonality | **34** | 14 | **0.004** |
| Climate | | | |
| Climate Occupancy | 29 | 19 | 0.149 |
| Maxent Niche Breadth | **36** | 12 | **0.001** |

Table S8. Ranking of 48 Allopolyploid plants with a single known diploid progenitor for 12 geographic and environmental range measures, as compared to their progenitor. Values indicate the number of comparisons in which the allopolyploid was the least extreme or most extreme in value, when compared to its one known progenitor. P reports the P-value from a Chi-Square test for each variable. Bold numbers indicate statistically significant differences from the null expectation of equal distribution, assessed at the Bonferroni adjusted P-value of 0.0042.

Table S9

|  | Allopolyploid Rank | |  |
| --- | --- | --- | --- |
|  | Least Extreme | Most Extreme | P |
| Geography | | | |
| Maximum latitude | 34 | 15 | 0.007 |
| Mean latitude | **37** | 12 | **< 0.001** |
| Latitude range | **38** | 11 | **0.001** |
| Area of Occupancy | 28 | 21 | 0.317 |
| Temperature | | | |
| Maximum | 23 | 26 | 0.668 |
| Minimum | **40** | 9 | **< 0.001** |
| Annual Range | **38** | 11 | **< 0.001** |
| Precipitation | | | |
| Maximum | 33 | 16 | 0.015 |
| Minimum | 26 | 23 | 0.668 |
| Seasonality | **35** | 14 | **0.003** |
| Climate | | | |
| Climate Occupancy | 32 | 17 | 0.032 |
| Maxent Niche Breadth | **39** | 10 | **< 0.001** |

Table S9. Ranking of 49 Allopolyploid seed plants with a single known progenitor for 12 geographic and environmental range measures, as compared to their progenitor. Values indicate the number of comparisons in which the allopolyploid was the least extreme or most extreme in value, when compared to its one known progenitor. P reports the P-value from a Chi-Square test for each variable. Bold numbers indicate statistically significant differences from the null expectation of equal distribution, assessed at the Bonferroni adjusted P-value of 0.0042.

Table S10

|  | Allopolyploid Rank | |  |
| --- | --- | --- | --- |
|  | Least Extreme | Most Extreme | P |
| Geography | | | |
| Maximum latitude | 32 | 13 | 0.005 |
| Mean latitude | **37** | 8 | **< 0.001** |
| Latitude range | **34** | 11 | **0.001** |
| Area of Occupancy | 24 | 21 | 0.655 |
| Temperature | | | |
| Maximum | 20 | 26 | 0.456 |
| Minimum | **36** | 9 | **< 0.001** |
| Annual Range | **35** | 10 | **< 0.001** |
| Precipitation | | | |
| Maximum | 29 | 16 | 0.053 |
| Minimum | 20 | 25 | 0.456 |
| Seasonality | **32** | 13 | **0.005** |
| Climate | | | |
| Climate Occupancy | 28 | 17 | 0.101 |
| Maxent Niche Breadth | **35** | 10 | **< 0.001** |

Table S10. Ranking of 45 Allopolyploid seed plants with a single known diploid progenitor for 12 geographic and environmental range measures, as compared to their progenitor. Values indicate the number of comparisons in which the allopolyploid was the least extreme or most extreme in value, when compared to its one known progenitor. P reports the P-value from a Chi-Square test for each variable. Bold numbers indicate statistically significant differences from the null expectation of equal distribution, assessed at the Bonferroni adjusted P-value of 0.0042.
